# Transforming Growth Factor-β-Activated Kinase 1 Is Required for Human FcγRIIIb-Induced Neutrophil Extracellular Trap Formation

**DOI:** 10.3389/fimmu.2016.00277

**Published:** 2016-07-18

**Authors:** Omar Rafael Alemán, Nancy Mora, Ricarda Cortes-Vieyra, Eileen Uribe-Querol, Carlos Rosales

**Affiliations:** ^1^Departamento de Inmunología, Instituto de Investigaciones Biomédicas, Universidad Nacional Autónoma de México, Ciudad de México, Mexico; ^2^División de Estudios de Posgrado e Investigación, Facultad de Odontología, Universidad Nacional Autónoma de México, Ciudad de México, Mexico

**Keywords:** immunoglobulin, immunoreceptor, inflammation, neutrophil, DNA, TAK1, ERK

## Abstract

Neutrophils (PMNs) are the most abundant leukocytes in the blood. PMN migrates from the circulation to sites of infection where they are responsible for antimicrobial functions. PMN uses phagocytosis, degranulation, and formation of neutrophil extracellular traps (NETs) to kill microbes. Several stimuli, including bacteria, fungi, and parasites, and some pharmacological compounds, such as Phorbol 12-myristate 13-acetate (PMA), are efficient inducers of NETs. Antigen–antibody complexes are also capable of inducing NET formation. Recently, it was reported that FcγRIIIb cross-linking induced NET formation similarly to PMA stimulation. Direct cross-linking of FcγRIIA or integrins did not promote NET formation. FcγRIIIb-induced NET formation presented different kinetics from PMA-induced NET formation, suggesting differences in signaling. Because FcγRIIIb also induces a strong activation of extracellular signal-regulated kinase (ERK) and nuclear factor Elk-1, and the transforming growth factor-β-activated kinase 1 (TAK1) has recently been implicated in ERK signaling, in the present report, we explored the role of TAK1 in the signaling pathway activated by FcγRIIIb leading to NET formation. FcγRIIIb was stimulated by specific monoclonal antibodies, and NET formation was evaluated in the presence or absence of pharmacological inhibitors. The antibiotic LL Z1640-2, a selective inhibitor of TAK1 prevented FcγRIIIb-induced, but not PMA-induced NET formation. Both PMA and FcγRIIIb cross-linking induced phosphorylation of ERK. But, LL Z1640-2 only inhibited the FcγRIIIb-mediated activation of ERK. Also, only FcγRIIIb, similarly to transforming growth factor-β-induced TAK1 phosphorylation. A MEK (ERK kinase)-specific inhibitor was able to prevent ERK phosphorylation induced by both PMA and FcγRIIIb. These data show for the first time that FcγRIIIb cross-linking activates TAK1, and that this kinase is required for triggering the MEK/ERK signaling pathway to NETosis.

## Introduction

Neutrophils are innate immune cells that migrate from the circulation to sites of inflammation or infection. Classically, neutrophils are considered the first line of defense since they are the first cells to appear at the affected site, and they display important antimicrobial functions (1). Neutrophils use phagocytosis, degranulation, and formation of neutrophil extracellular traps (NETs) to kill microbes (2, 3). NETs are formed through a unique cell death program named “NETosis” that involves first degradation of the nuclear membrane and chromatin expansion into the cytosol, while the cell membrane remains intact. Later, 3 or 4 h after stimulation, the cell membrane breaks, and the chromatin fibers get expelled outside the cell, creating a net-like structure. NET fibers are composed of chromatin covered with histones (4) and antimicrobial proteins derived from the neutrophil granules, such as the bactericidal/permeability-increasing protein (BPI), elastase, myeloperoxidase, lactoferrin, and metalloprotease 9 (2, 5). NETs prevent further spread of pathogens because they function as a physical barrier where microorganisms get trapped and because they bring antimicrobial proteins in close proximity of pathogens. Thus, NETs can kill microorganisms extracellularly and independently of phagocytosis (6).

Human neutrophils express constitutively two IgG antibody receptors: FcγRIIa (CD32a) and FcγRIIIb (CD16b) (7). FcγRIIa consists of a single polypeptide chain bearing an ITAM on its cytoplasmic domain (8). This ITAM confers on FcγRIIa the ability to initiate signaling events that regulate cell responses, including phagocytosis, cytokine production, and antibody-dependent cell-mediated cytotoxicity (9). FcγRIIIb is present exclusively on neutrophils, and it is a glycophosphatidylinositol (GPI)-linked receptor, lacking transmembrane and cytoplasmic domains (10). The signaling mechanism for this receptor is still unknown, since possible signaling molecules directly associated with it remain unidentified. However, several reports show that FcγRIIIb can initiate signaling events leading to various cell responses including increase in calcium concentration (11), activation of integrins (12), and activation of NF-κB (13, 14).

FcγRIIIb cross-linking induced efficient NET formation similarly to Phorbol 12-myristate 13-acetate (PMA) stimulation (15). This NET formation was dependent on NADPH-oxidase and extracellular signal-regulated kinase (ERK) activation (15). But, the mechanism linking FcγRIIIb to ERK is not known. Previously, we reported that FcγRIIIb cross-linking led to activation of NF-κB (13); while others have reported that transforming growth factor-β-activated kinase 1 (TAK1) was associated to the IκB kinase complex, both in the nucleus and cytoplasm of human neutrophils favoring NF-κB activation (16). More recently, we also found that FcγRIIIb induced a robust activation of ERK and also of the transcription factor Elk-1 (17), but we could not identify the molecule responsible for ERK activation. Similarly, others have reported that, in human neutrophils, TAK1 acted upstream of MEK (ERK kinase) and ERK signaling pathway (18, 19). Thus, in this report, we explored the possibility that TAK1 is functionally coupled to FcγRIIIb leading to NETosis *via* ERK activation. FcγRIIIb was stimulated by specific monoclonal antibodies, and the NET formation was evaluated in the presence or absence of pharmacological inhibitors. The antibiotic LL Z1640-2, a selective inhibitor of TAK1 prevented FcγRIIIb-induced, but not PMA-induced NET formation. Both PMA and FcγRIIIb cross-linking induced phosphorylation of ERK. But, LL Z1640-2 only inhibited the FcγRIIIb-mediated activation of ERK. Also, a MEK-specific inhibitor was able to prevent ERK phosphorylation induced by both PMA and FcγRIIIb. These data show for the first time that FcγRIIIb cross-linking activates TAK1, and that, this kinase is required for triggering the MEK/ERK signaling pathway to NETosis.

## Materials and Methods

### Neutrophils

Neutrophils were isolated from the peripheral blood collected from adult healthy volunteers following a protocol that was approved by the Bioethics Committee at Instituto de Investigaciones Biomédicas – UNAM. All volunteers provided a written informed consent for their blood donation. The procedure for neutrophil isolation was exactly as previously described (14).

### Reagents

Bovine serum albumin (BSA) was from F. Hoffmann-La Roche Ltd. (Mannheim, Germany). Piceatannol, a spleen tyrosine kinase (Syk) inhibitor was from Acros Organics (NJ, USA). PD98059 and U0126, MEK (ERK kinase) inhibitors were obtained from New England Biolabs (Beverly, MA, USA) and from Promega (Madison, WI, USA), respectively. The antibiotic LL Z1640-2 [also known as (5Z)-7-Oxozeaenol; cas 66018-38-0] (catalog no. sc-202055) was from Santa Cruz Biotechnology (Santa Cruz, CA, USA). GÖ6983, a protein kinase C (PKC) inhibitor, SB 203580, a p38 MAP kinase inhibitor (catalog number 559389), and 3-(1-methyl-1H-indol-3-yl-methylene)-2-oxo-2,3-dihydro-1H-indole-5-sulfonamide (iSyk), another Syk inhibitor (catalog no. 574711) were from Calbiochem/EMD Millipore (Billerica, MA, USA). Recombinant Human TGF-β1 (catalog No. 100-21) was from Peprotech (Rocky Hill, NJ, USA). The cOmplete™ protease inhibitor cocktail (catalog No. 11697498001) and *PhosSTOP*™ phosphatase inhibitor cocktail (catalog No. 04906845001) were from Roche Diagnostics (Basel, Switzerland). PMA and all other chemicals were from Sigma Aldrich (St. Louis, MO, USA). The following antibodies were used: anti-human FcγRI (CD64) mAb 32.2 (ATCC® HB-946™) and anti-human FcγRIIa (CD32a) mAb IV.3 (20) (ATCC® HB-217™) were from American Type Culture Collection (Manassas, VA, USA). The anti-human FcγRIIIb (CD16b) mAb 3G8 (21) was donated by Dr. Eric J. Brown (University of California in San Francisco, San Francisco, CA, USA). The anti-β1 integrin mAb TS2/16 was donated by Martin Hemler (Dana Farber Cancer Research Institute, Boston, MA, USA). Monoclonal antibodies were purified as previously described (15). Rabbit polyclonal anti-ERK 1 (catalog no. sc-94), rabbit polyclonal anti-phospho-ERK 1/2 (pTyr204) (catalog no. sc-101761), and rabbit polyclonal anti-glyceraldehyde 3-phosphate dehydrogenase (GAPDH) (catalog no. sc-25778) were from Santa Cruz Biotechnology (Santa Cruz, CA, USA). F(ab′)_2_ fragment goat anti-mouse IgG (catalog No. 115-006-072) was from Jackson Immuno Research Laboratories Inc. (West Grove, PA, USA). HRP-conjugated F(ab′)_2_ goat anti-mouse IgG (catalog No. 0855572) and HRP-conjugated F(ab′)_2_ goat anti-rabbit IgG (catalog No. 0855686) were from MP Biomedicals (Santa Ana, CA, USA). Rabbit polyclonal anti-phospho-TAK1 (T187) (catalog No. ab192443) was from Abcam plc. (Cambridge, UK).

### NET Formation Kinetics

Neutrophil extracellular trap formation was quantified by detecting DNA release spectrophotometrically with the DNA-binding dye SYTOX® Green (22–24). For PMA stimulation, neutrophils were resuspended at 1 × 10^6^ cell/ml in RPMI-1640 medium (Gibco®; Grand Island, NY, USA) containing 500 nM SYTOX® Green (Molecular Probes, Inc.; Eugene, OR, USA), and 100 μl of this cell suspension (1 × 10^5^ PMN) were added to each well of the 96-well plate. The plate was then incubated at 35°C for 20 min in a microplate reader model Synergy HT from BioTek Instruments (Winooski, VT, USA). Next, 20 μl of 120 nM PMA dissolved in the same RPMI/SYTOX medium were added to each well for a final concentration of 20 nM. After that, the plate was incubated for up to 4 h, reading the fluorescence from the bottom of the plate, using the 485 nm excitation and 528 emission filters, every 5 min. For FcγR stimulation, neutrophils were resuspended at 0.5 × 10^7^ cell/ml in RPMI/SYTOX medium containing 10 μg/ml of the corresponding anti-FcγR antibody and incubated in ice for 20 min. After one wash in PBS, cells were resuspended in the same volume of RPMI/SYTOX medium, and 20 μl of this cell suspension (1 × 10^5^ PMN) were added to each well of the 96-well plate. The plate was then incubated at 35°C for 20 min in a microplate reader. Next, 100 μl of 45 μg/ml goat anti-mouse IgG in RPMI/SYTOX medium were added to each well (final concentration 37.5 μg/ml). Finally, the plate was incubated for up to 4 h, reading the fluorescence every 5 min. For TAK1 inhibition, cells were treated with 10 nM LL Z1640-2 for 30 min before stimulation.

### Neutrophil Stimulation

PMNs were stimulated by cross-linking Fc receptors with specific mAbs as follows: PMN were resuspended in PBS at 1 × 10^7^ cells/ml, and 200 μl of the cell suspension were placed in Eppendorf tubes. The corresponding mAb was then added at 10 μg/ml, and the cells were incubated on ice for 30 min. Next, cells were washed twice with 500 μl of PBS. Receptor cross-linking was then induced by resuspending the cells in 100 μl of PBS containing 37 μg/ml of F(ab′)_2_ goat anti-mouse IgG and incubating them at 37°C for 15 min. For PMN stimulation with PMA or TGF-β, PMN were incubated at 37°C for 15 min with 20 nM PMA or 5 ng/ml TGF-β. In assays where pharmacological inhibitors were used, PMN were pretreated with 10 nM LLZ 640-2 or only with the solvent dimethyl sulfoxide (DMSO) on ice for 30 min before adding the first mAb.

### Protein Extraction and Western Blotting

Total protein extracts were obtained by lysing the cells in cold RIPA lysis buffer (150 mM NaCl, 5 mM EDTA, 50 mM Hepes, 0.5% sodium deoxycholate, 1% Non-idet P-40, 50 mM NaF, and 1 mM sodium orthovanadate, pH 7.5) supplemented with 1× protease inhibitor cocktail and 1× phosphatase inhibitor cocktail, which were added just before lysing the cells. Cell lysates were incubated on ice for 20 min, then cleared by centrifugation, and proteins resolved on SDS 10% PAGE. Proteins were then electrotransfered onto polyvinylidine fluoride (PVDF) membranes (Immobilon-P; Millipore, Bedford, MA, USA). Membranes were incubated in blocking buffer (1% BSA, 5% non-fat dry milk) (Carnation; Nestle, Glendale, CA, USA) and 0.1% Tween 20 in Tris-buffered saline (TBS: 50 mM Tris-HCl, 150 mM NaCl, pH = 7.5) overnight at 4°C. Membranes were subsequently probed with the corresponding antibody in blocking buffer for 1 h at room temperature. Anti-phospho-ERK 1 (1/1000 dilution) or anti-phospho TAK1 (1/2000 dilution). Membranes were washed with TBS-Tween six times and incubated with a 1/3000 dilution of HRP-conjugated F(ab′)_2_ goat anti-rabbit IgG o for 1 h at room temperature. After washing six more times, the membrane was developed with Immobilon Western chemiluminescent HRP substrate (catalog No. WBKLS0100) from EMD Millipore (Billerica, MA, USA) according to the manufacturer’s instructions. Afterward, membranes were stripped with 0.2 M NaOH and reprobed with anti-ERK 1 (1/2000 dilution) or anti-GAPDH (1/1000 dilution) to assess protein loading in PAGE gels.

### Statistical Analysis

Quantitative data were expressed as mean ± SEM. Single variable data were compared by paired-sample Student’s *t*-tests using the computer program KaleidaGraph® version 3.6.2 for Mac (Synergy Software; Reading, PA, USA). Differences were considered statistically different at a value *p* < 0.05.

## Results

### FcγRIIIb-Mediated NETosis Presents a Different Kinetics from PMA-Induced NETosis

**Figure 1:**
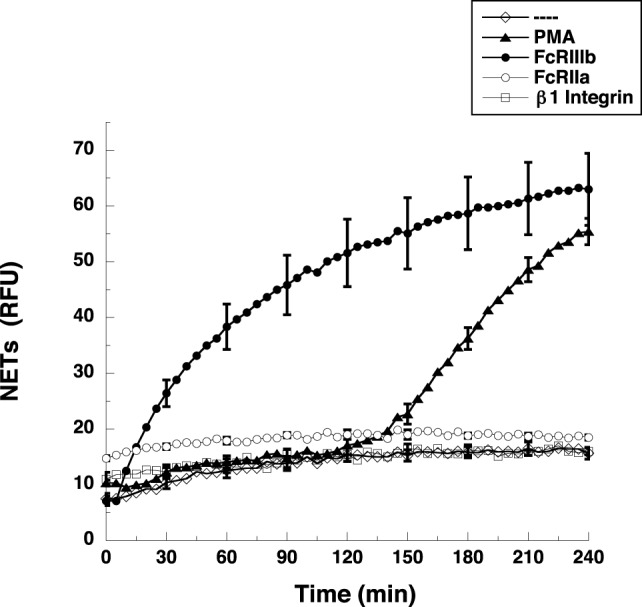
**FcγRIIIb induces NET formation faster than PMA**. Human neutrophils were left untreated (---), or were stimulated with 20 nM phorbol 12-myristate 13-acetate (PMA), or by cross-linking FcγRIIIb with mAb 3G8, or by cross-linking FcγRIIa with mAb IV.3, or by cross-linking β1 integrins with mAb TS2/16, and then incubated for 4 h. The relative amount of NETs was estimated from SYTOX^®^ Green fluorescence in relative fluorescent units (RFU) every 5 min. Data are mean ± SEM of three experiments done in tetraplicates.

Most studies on NETs have used PMA, a potent activator of PKC, to induce the formation of NETs (2). Direct cross-linking of FcγRIIIb also leads to a robust activation of NET formation (15). However, the kinetics of these responses is different. When human neutrophils were stimulated by PMA, NETosis (5) is observed as a late event with NETs (extracellular DNA fibers) detected after 2.5 h of stimulation (Figure 1). Complete NET formation was seen, as previously described, by 4 h after stimulation (Figure 1). In contrast, stimulation of FcγRIIIb with mAb 3G8, induced NETosis with a much faster kinetics. By 30 min after receptor cross-linking, NETs could already be detected (Figure 1). By 2 h, about half of the total amount of NETs had already been formed, and by 4 h, NETs reached a level similar to that induced by PMA (Figure 1). In order to confirm that the mAb 3G8 (IgG1) was specifically targeting (cross-linking) FcγRIIIb, neutrophils were also stimulated by the isotypic control antibody TS2/16 (IgG1) that binds to β1 integrins, and by the mAb IV.3 (IgG2b) that binds FcγRIIa. Neither mAb IV.3 nor mAb TS2/16 induced NET formation (Figure 1), strengthening the point that FcγRIIIb is the receptor responsible for induction of NETosis. These data indicated that cross-linking FcγRIIIb is an efficient stimulus for NET formation with a faster response than the one induced by PMA. This difference in response kinetics led us to explore the signaling pathway from FcγRIIIb to NETosis.

### TAK1 Is Involved in FcγRIIIb-Mediated NETosis

**Figure 2:**
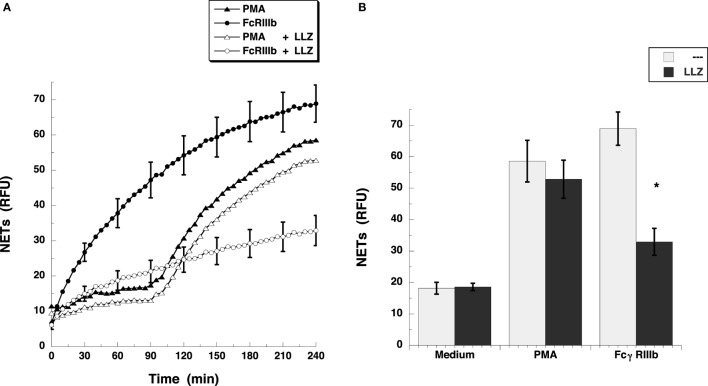
**TAK1 is required for FcγRIIIb-induced NET formation**. **(A)** human neutrophils were stimulated with 20 nM phorbol 12-myristate 13-acetate (PMA), or by cross-linking FcγRIIIb with mAb 3G8, and then incubated for 4 h. Some neutrophils were previously treated with 10 nM LL Z1640-2 (LLZ), a selective inhibitor of TAK1 (white symbols), or with only the solvent (DMSO) (black symbols). The relative amount of NETs was estimated from SYTOX^®^ Green fluorescence in relative fluorescent units (RFU) every 5 min. Data are mean ± SEM of three experiments done in tetraplicates. **(B)** human neutrophils were previously treated with solvent alone (---) or with the 10 nM LL Z1640-2 (LLZ), before stimulating with nothing (medium), 20 nM PMA, or by cross-linking FcγRIIIb, and then incubated for 4 h. The relative amount of NETs was estimated from SYTOX^®^ Green fluorescence in relative fluorescent units (RFU) at 4 h after stimulation. Data are mean ± SEM of three experiments done in tetraplicates. Asterisks denote conditions that are statistically different from control (*p* < 0.0003).

Others and we have seen that the MEK/ERK signaling pathway is required for both PMA- (25) and FcγRIIIb-induced NETosis (15, 23). Because the transforming growth factor-β-activated kinase 1 (TAK1) is a known activator of MAP kinase signaling pathways in various immune cells (26), and in human neutrophils, TAK1 was also reported to act upstream of ERK (18), we explored the possibility that TAK1 is involved in FcγRIIIb-mediated NETosis. The antibiotic LL Z1640-2, a selective inhibitor of TAK1 prevented FcγRIIIb-induced NET formation (Figure 2A), but not PMA-induced NET formation (Figure 2A). The inhibitory effect was maximum at 4 h after stimulation when the amount of NETs from FcγRIIIb-stimulated neutrophils was reduced by half (Figure 2B). This result indicated for the first time that indeed TAK1 is involved in NET formation after cross-linking FcγRIIIb.

**Figure 3:**
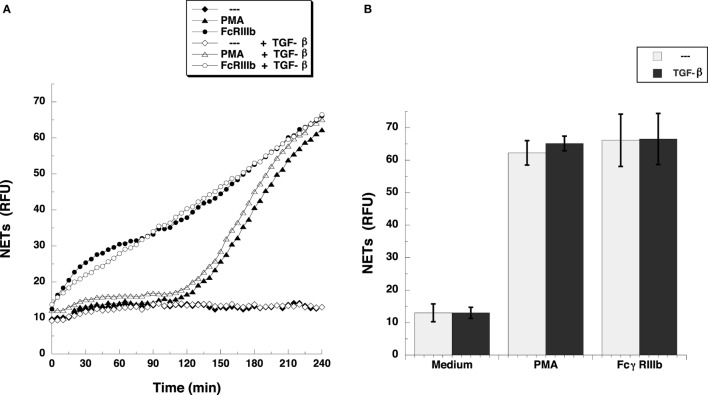
**TGF-β does not affect FcγRIIIb-induced NET formation**. **(A)** human neutrophils were left untreated (---), or were stimulated with 20 nM phorbol 12-myristate 13-acetate (PMA), or by cross-linking FcγRIIIb, and then incubated for 4 h. Some neutrophils were previously treated with 5 ng/ml transforming growth factor-β (TGF-β) (white symbols) or with only the solvent (DMSO) (black symbols). The relative amount of NETs was estimated from SYTOX^®^ Green fluorescence in relative fluorescent units (RFU) every 5 min. Data are mean ± SEM of three experiments done in tetraplicates. **(B)** human neutrophils were previously treated with solvent alone (---) or with 5 ng/ml TGF-β, before stimulating with nothing (Medium), 20 nM PMA, or by cross-linking FcγRIIIb, and then incubated for 4 h. The relative amount of NETs was estimated from SYTOX^®^ Green fluorescence in relative fluorescent units (RFU) at 4 h after stimulation. Data are mean ± SEM of three experiments done in tetraplicates.

**Figure 4:**
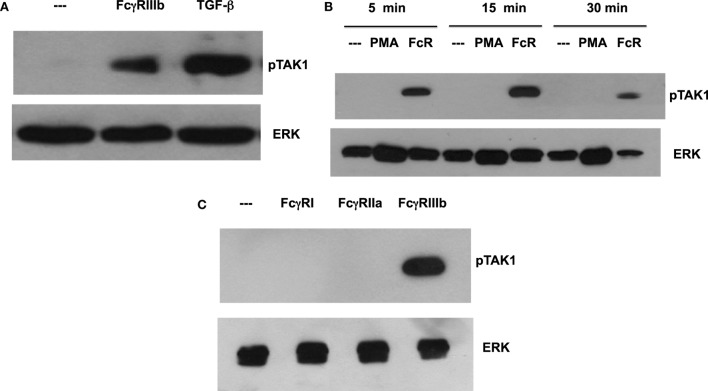
**FcγR-cross-linking induces activation of TAK1**. Human neutrophils were left untreated (---), or **(A)** were stimulated with 5 ng/ml transforming growth factor-β (TGF-β), or by cross-linking FcγRIIIb for 15 min. **(B)** neutrophils were also stimulated with 20 nM phorbol 12-myristate 13-acetate (PMA), or by cross-linking FcγRIIIb (FcR) for 5, 15, and 30 min. **(C)** neutrophils were also stimulated by cross-linking FcγRI with mAb 32.2, by cross-linking FcγRIIa with mAb IV.3, or by cross-linking FcγRIIIb with mAb 3G8 for 15 min. Cell lysates were prepared after stimulation. Proteins were resolved by SDS-PAGE, and then Western blotted for phosphorylated-TAK1 (pTAK1) (upper panel) or for total ERK (lower panel). Data are representative of three separate experiments.

Transforming growth factor-β-activated kinase 1 was initially identified as a regulator of MAPK in response to TGF-β (27), thus, we explored whether TGF-β could have an effect on NETosis. Treatment of neutrophils with TGF-β did not change the kinetics nor the amount of NET formation induced either by FcγRIIIb cross-linking or PMA stimulation (Figure 3). This lack of effect on NETosis was not due to failure of TGF-β to activate TAK1. Neutrophils treated with TGF-β presented a robust phosphorylation of TAK1 (Figure 4A) indicating that the axis TGF-β/TAK1 was functional in these cells. Moreover, cross-linking of FcγRIIIb also led to phosphorylation of TAK1 (Figure 4A). This phosphorylation in Thr-187 is indicative of activation of TAK1 (28). The FcγRIIIb-mediated phosphorylation of TAK1 was detectable at 5 min, reached a maximum at 15 min, and was almost gone by 30 min after receptor cross-linking (Figure 4B). Thus, this time was used in all other experiments to detect TAK1 phosphorylation. Opposite to this result, treatment with PMA did not induce any phosphorylation of TAK1 (Figure 4B).

Human neutrophil expresses constitutively two low-affinity Fcγ receptors, FcγRIIa and FcγRIIIb, and after interferon-γ, they can upregulate FcγRI. Previously, it has been reported that FcγRIIIb is the receptor responsible for NET formation (15, 23). Therefore, we explored the possibility that each of these Fc receptors could activate TAK1 after cross-linking each receptor with the corresponding specific monoclonal antibody. Treating the cells with monoclonal antibody 32.2 (anti-FcγRI) did not induce TAK1 phosphorylation (Figure 4C). This was an expected result since FcγRI is not expressed in resting neutrophils. Similarly, cross-linking with monoclonal antibody IV.3 (anti-FcγRIIa) also did not cause any TAK1 phosphorylation (Figure 4C). In contrast, cross-linking of FcγRIIIb with the monoclonal antibody 3G8 efficiently induced TAK1 phosphorylation (Figure 4). Together, these data suggested that, indeed, FcγRIIIb signaling in human neutrophils requires TAK1 activation for induction of NET formation.

### Syk Is Required for FcγRIIIb-Mediated TAK1 Activation

**Figure 5:**
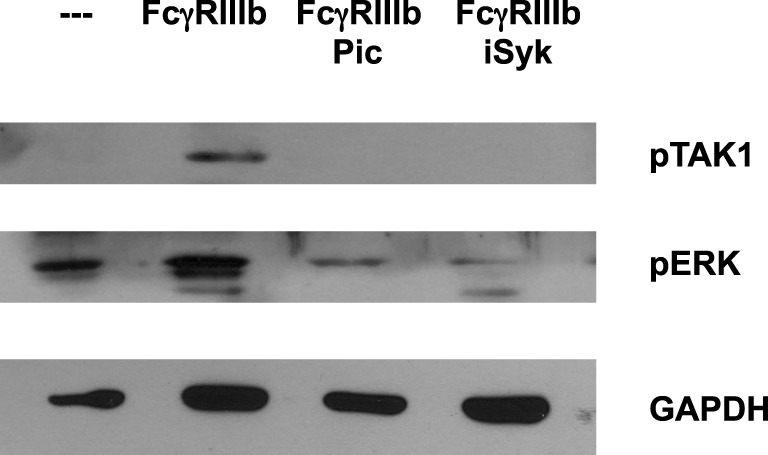
**Syk is required for FcγRIIIb-mediated TAK1 activation**. Human neutrophils were left untreated (---), or were stimulated by cross-linking FcγRIIIb for 15 min in the absence or presence of 50 μM Piceatannol (Pic) or 40 nM iSyk, both selective inhibitors of Syk. Cell lysates were prepared after stimulation. Proteins were resolved by SDS-PAGE and then Western blotted for phosphorylated-TAK1 (pTAK1) (upper panel), or for phosphorylated ERK (pERK) (middle panel), and for total glyceraldehyde 3-phosphate dehydrogenase (GAPDH) (lower panel). Data are representative of three separate experiments.

After establishing a role for TAK1 in FcγRIIIb-mediated NET formation, we explored a possible connection from FcγRIIIb to TAK1. Neutrophils were stimulated by FcγRIIIb cross-linking in the presence or absence of two Syk inhibitors. FcγRIIIb-induced TAK1 phosphorylation and also ERK 1 phosphorylation were efficiently blocked by both Syk inhibitors (Figure 5). This result suggested that FcγRIIIb connects to TAK1 activation *via* Syk.

### TAK1 Is Required for FcγRIIIb-Mediated ERK Activation

**Figure 6:**
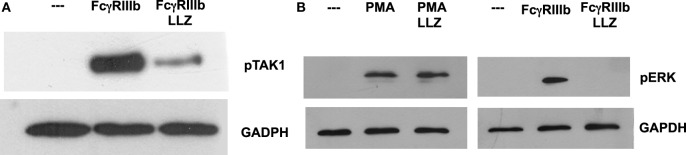
**TAK1 is required for FcγRIIIb-mediated ERK activation**. Human neutrophils were left untreated (---), or were stimulated by cross-linking FcγRIIIb for 15 min, or by 20 nM phorbol 12-myristate 13-acetate (PMA). Some neutrophils were previously treated with 10 nM LL Z1640-2 (LLZ), a selective inhibitor of TAK1. Cell lysates were prepared after stimulation. Proteins were resolved by SDS-PAGE, and then, Western blotted for **(A)** phosphorylated-TAK1 (pTAK1) (upper panel) and for total glyceraldehyde 3-phosphate dehydrogenase (GAPDH) (lower panel); or for **(B)** phosphorylated ERK (pERK) and total GAPDH (lower panel). Data are representative of three separate experiments.

Next, we explored the signaling pathway from TAK1 to ERK. Neutrophils were stimulated by PMA or FcγRIIIb cross-linking in the presence or absence of the TAK1 inhibitor, and ERK 1 activation was detected by Western blotting. First, we confirmed that LL Z1640-2 was inhibiting TAK1 phosphorylation (Figure 6A). Under the same conditions, PMA induced ERK phosphorylation (Figure 6B) as previously reported (15). This ERK phosphorylation was not affected by the TAK1 inhibitor (Figure 6B). In contrast, FcγRIIIb cross-linking also induced ERK phosphorylation, but this ERK phosphorylation was efficiently blocked by the TAK1 inhibitor (Figure 6B). This result strongly indicated that TAK1 activation is required for ERK activation after FcγRIIIb cross-linking, but not after PMA stimulation.

**Figure 7:**
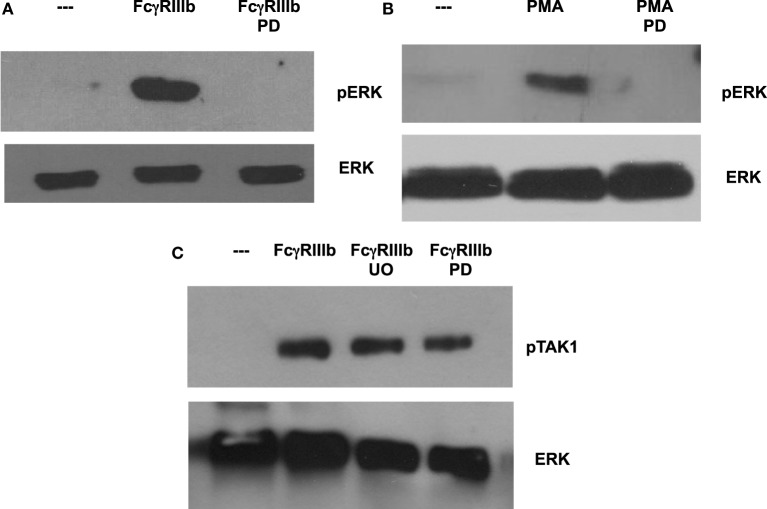
**MEK1 is required for FcγRIIIb-mediated ERK activation**. Human neutrophils were left untreated (---), or were stimulated by cross-linking FcγRIIIb for 15 min, or by 20 nM phorbol 12-myristate 13-acetate (PMA). Some neutrophils were previously treated with 50 μM PD98059 (PD) or with 50 μM UO126 (UO), both selective inhibitors of MEK. Cell lysates were prepared after stimulation. Proteins were resolved by SDS-PAGE, and then Western blotted for **(A,B)** phosphorylated ERK (pERK) (upper panel) and for total ERK (lower panel); or for **(C)** phosphorylated-TAK1 (pTAK1) and for total ERK (lower panel). Data are representative of three separate experiments.

In most situations, MEK activation leads to ERK activation, as the former phosphorylates the latter. In order to confirm that this is also the case in the case of FcγRIIIb- or PMA-induced NETosis, neutrophils were treated with the MEK inhibitor PD98059 prior to stimulation. As shown before, cross-linking of FcγRIIIb clearly activated ERK, and this activation was completely blocked by the MEK inhibitor (Figure 7A). Similarly, this MEK inhibitor also blocked ERK activation induced by PMA (Figure 7B). Neither PD98059 nor UO126, another potent MEK inhibitor, was able to block TAK1 activation induced by FcγRIIIb (Figure 7C). This last result confirms that TAK1 is upstream of MEK/ERK signaling module in the case of FcγRIIIb signaling. These data also suggest that the signaling pathways initiated by both FcγRIIIb and PMA converge at the level of PKC or MEK to activate ERK leading to downstream NETosis.

### p38 MAPK Is Not Required for FcγRIIIb-Mediated NET Formation

**Figure 8:**
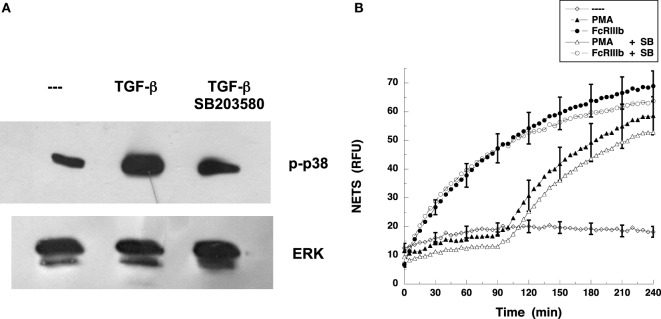
**p38 MAPK is not required for FcγRIIIb-mediated NET formation**. **(A)** Human neutrophils were left untreated (---) or were stimulated with 5 ng/ml transforming growth factor-β (TGF-β). Some neutrophils were previously treated with 100 nM SB 203580, a selective inhibitor of p38 MAPK. Cell lysates were prepared after stimulation. Proteins were resolved by SDS-PAGE, and then Western blotted for phosphorylated-p38 (p-p38) (upper panel) and for total ERK (lower panel). Data are representative of three separate experiments. **(B)** human neutrophils were left untreated (---), or were stimulated with 20 nm phorbol 12-myristate 13-acetate (PMA), or by cross-linking FcγRIIIb with mAb 3G8, and then incubated for 4 h. Some neutrophils were previously treated with 100 nM SB 203580 (SB) (open symbols). The relative amount of NETs was estimated from SYTOX^®^ Green fluorescence in relative fluorescent units (RFU) every 5 min. Data are mean ± SEM of three experiments done in tetraplicates.

Because it is well known that TAK1 functions upstream of p38 MAPK pathway rather than ERK (26, 29) in many cell types, we examined whether blockade of p38 MAPK affected FcγRIIIb-induced NET formation. The specific p38 MAPK inhibitor SB203580 blocked phosphorylation of p38 MAPK induced by TGF-β (Figure 8A). As expected, neutrophils treated with PMA in the presence of SB203580 produced NETs as efficiently as the neutrophils with no inhibition of p38 MAPK (Figure 8B). Similarly, inhibition of p38 MAPK did not affect NET formation induced by cross-linking FcγRIIIb (Figure 8B). These data strongly suggest that FcγRIIIb activates TAK1 to connect with the MEK/ERK pathway in order to activate NET formation.

## Discussion

The MAP3K, TAK1, is activated by different stimuli including cytokines such as tumor necrosis factor (TNF), interleukin (IL)-1, and IL-18, or TLR ligands such as LPS in various cell types (26, 30–34) and participates in activating several signaling pathways. In this study, we report for the first time that, in human neutrophils, TAK1 can also be activated in response to stimulation of antibody receptor FcγRIIIb. We also show that TAK1 is required for induction of NETosis by this receptor *via* the MEK/ERK signaling cascade.

Neutrophil activation is required for the initiation of the several defense mechanisms, including phagocytosis, respiratory burst, release of various microbicidal molecules by degranulation (35), and the formation of NETs (3). Many pathogens, including virus, bacteria, fungi, and parasites are known to induce NET formation (6). These microorganisms must be recognized by pattern recognition receptors (PRRs) such as TLRs. In fact, TLR-4 has been identified as an important receptor for NET formation (36–38). In addition, receptors for the Fc portion of antibody molecules have recently been identified as potent inducers of NET formation. In particular, the receptor for IgA FcαRI (CD89) (39) and the receptor for IgG FcγRIIIb (CD16b) (15, 23) are the only Fc receptors known to induce NETosis.

FcγRIIIb is present exclusively on human neutrophils, and it is a GPI-linked receptor, lacking transmembrane and cytoplasmic domains (10). Despite the fact that the initial signaling mechanism for this receptor remains to be described, it is clear that it can activate several signaling pathways leading to various cell responses including increase in calcium concentration (11), activation of integrins (12), activation of the transcription factors NF-κB (13) and Elk-1 (17), and induction of NET formation (15, 23). In our previous publication, we described that FcγRIIIb can activate ERK, and this activation is important for NET formation (15). However, we could not identify how the MEK/ERK signaling cascade was engaged. Here, we now report for the first time, as far as we know, that the transforming growth factor-β-activated kinase 1 (TAK1) is activated upon FcγRIIIb engagement, and that this kinase is required both for NET formation and MEK/ERK activation. Our findings are similar to those reported for chemoattractant and growth factor stimulation of neutrophils where TAK1 is also activated and acts upstream of the MEK/ERK pathway (19). Still, the manner in which FcRIIIb activates TAK1 remains elusive. Possible activators include Syk or TRAF6. We addressed the involvement of Syk by blocking its activity with two different specific inhibitors. Both Piceatannol and iSyk prevented activation (phosphorylation) of both TAK1 and ERK. These data clearly indicate that Syk functions upstream of TAK1 after FcγRIIIb engagement. However, how this receptor lacking a cytoplasmic tail can connect with Syk remains an unsolved problem for future studies.

**Figure 9:**
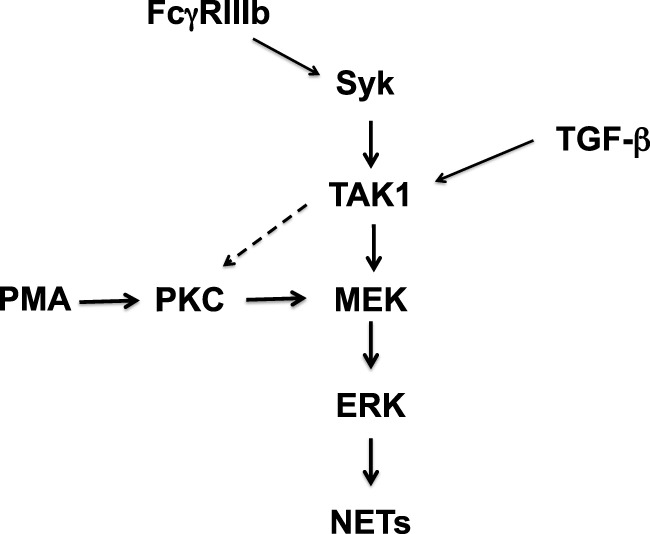
**Model for signaling in neutrophils to induce NETosis**. In human neutrophils, cross-linking FcγRIIIb leads to activation of spleen tyrosine kinase (Syk) and transforming growth factor-β-activated kinase 1 (TAK1). TAK1 is then required for activation of ERK kinase (MEK) and extracellular signal-regulated kinase (ERK). Activated ERK contributes to the formation of neutrophil extracellular traps (NETs). Phorbol 12-myristate 13-acetate (PMA) can directly activate protein kinase C (PKC), which in turn leads to activation of the MEK/ERK pathway. These kinases finally promote NET formation. FcγRIIIb-induced NET formation also depends on PKC (15). Thus, TAK1 might also be able to activate PKC (dashed arrow), but this remains to be demonstrated. Transforming growth factor-β (TGF-β) is a potent activator of TAK1. However, this activation does not seem to have any effect of NET formation induced by PMA or FcγRIIIb.

Although, both stimuli PMA and FcγRIIIb cross-linking initiate signaling that seems to converge at the level of MEK (Figure 9), an important difference in NETosis induced by PMA or by FcγRIIIb was found in this study. PMA release of DNA fibers was detected at later times just as described before (2, 40), more than 2½ h after stimulation, and reached a maximum around 4 h (Figure 1). In contrast, FcγRIIIb-induced NETosis liberated DNA fibers rapidly in less than 1 h (Figure 1). Mechanistically, we do not know the reason for this difference, but it is possible that another pathway in addition to the ERK pathway is involved. Previously, Syk was also found to participate in NET formation induced by insoluble immune complexes (23) and by PMA (23). We also found that inhibition of Syk by Piceatannol blocked the release of NETs induced either by PMA or by FcγRIIIb (15). In addition, we have observed inhibition of FcγRIIIb-mediated TAK1 phosphorylation by Piceatannol and by iSyk (Figure 5). This suggests as mentioned above that Syk is required for TAK1 activation to deliver a signal for NET formation after FcγRIIIb engagement. Yet, activation of Syk by PMA has also been previously described in neutrophils. PMA induced PKC-dependent phosphorylation of Syk (41). However, we do not think that this pathway is involved in this case because inhibition of PKC did not prevent FcγRIIIb-induced TAK1 phosphorylation (our unpublished data). Thus, TAK1 acts downstream of FcγRIIIb and upstream (or independently) of PKC (Figure 9). In contrast, inhibition of PKC leads to reduced FcγRIIIb-induced NET formation (15). Hence, it would seem that TAK1 connects to PKC for activation of the MEK/ERK signaling cascade. In support of this idea, another receptor has been recently reported to activate Syk and TAK1 together with PKC. The innate decoy receptor CEACAM3, also exclusively expressed by human neutrophils, triggers a Syk-, PKCδ-, and TAK1-dependent signaling cascade that results in activation of NF-κB (42). In another even more recent report, TAK1 was clearly shown to activate the MEK/ERK pathway (19). Unfortunately, in this study, the involvement of PKC was not investigated. Whether TAK1 connects to MEK directly of *via* PKC remains unsolved (Figure 9). Also, the difference in kinetics for NET formation might be due, at least in part, to the selective activation of TAK1 by FcγRIIIb (Figure 9). This idea is attractive, since, when neutrophils were treated with TGF-β, a stronger phosphorylation of TAK1 was detected (Figure 4A). Yet, no difference in NET formation was observed in cells pretreated with TGF-β. The mechanism responsible for the faster kinetics in FcγRIIIb-mediated NET formation remains to be elucidated.

In several cell types, TAK1 functions upstream of p38 MAPK pathway rather than ERK (26, 29). In contrast, in human neutrophils, it has been reported that chemotactic and growth factors induce TAK1 activation leading to the MEK/ERK pathway independently of p38 MAPK (19). In the case of FcγRIIIb-induced NET formation, we also found that inhibition of p38 MAPK with the inhibitor SB203580 did not affect NETosis (Figure 8B). Thus, our data also support the hypothesis that, in human neutrophils, TAK1 connects to MEK/ERK and not to p38 MAPK or JNK.

In conclusion, to our knowledge, this is the first demonstration that TAK1 can be activated by FcγRIIIb in human neutrophils, and that this kinase is required for triggering the MEK/ERK signaling pathway to NETosis.

## Author Contributions

OA performed most of the experiments for NET formation and analyzed the data. NM prepared the cells and performed Western blots. RC-V generated some of the Western blot data. EU-Q helped with the first draft, performed the statistical analysis, organized the references, and prepared all figures. CR designed the research, mentored all other authors, and wrote the final version of the paper.

## Conflict of Interest Statement

The authors declare that the research was conducted in the absence of any commercial or financial relationships that could be construed as a potential conflict of interest.
